# Study Protocol: Screening and Treatment of Alcohol-Related Trauma (START) – a randomised controlled trial

**DOI:** 10.1186/1472-6963-12-371

**Published:** 2012-10-29

**Authors:** Rama Jayaraj, Mahiban Thomas, David Kavanagh, Peter d’Abbs, Luke Mayo, Valerie Thomson, Carolyn Griffin, Tricia Nagel

**Affiliations:** 1 Wellbeing and Preventable Chronic Diseases Division, Menzies School of Health Research and School of Environmental and Life Sciences, Charles Darwin University, Darwin, Northern Territory, Australia; 2 School of Environmental and Life Sciences, Charles Darwin University, Darwin, Northern Territory, Australia; 3 Department of Head and Neck Surgery, Royal Darwin Hospital, Darwin, Northern Territory, Australia; 4 Institute of Health & Biomedical Innovation and School of Psychology and Counselling, Queensland University of Technology, Brisbane, Australia

**Keywords:** Facial trauma, Indigenous Australians or Aboriginal and Torres Strait Islanders, Alcohol related injury, Culturally appropriate intervention

## Abstract

**Background:**

The incidence of mandibular fractures in the Northern Territory of Australia is very high, especially among Indigenous people. Alcohol intoxication is implicated in the majority of facial injuries, and substance use is therefore an important target for secondary prevention. The current study tests the efficacy of a brief therapy, Motivational Care Planning, in improving wellbeing and substance misuse in youth and adults hospitalised with alcohol-related facial trauma.

**Methods and design:**

The study is a randomised controlled trial with 6 months of follow-up, to examine the effectiveness of a brief and culturally adapted intervention in improving outcomes for trauma patients with at-risk drinking admitted to the Royal Darwin Hospital maxillofacial surgery unit. Potential participants are identified using AUDIT-C questionnaire. Eligible participants are randomised to either Motivational Care Planning (MCP) or Treatment as Usual (TAU). The outcome measures will include quantity and frequency of alcohol and other substance use by Timeline Followback. The recruitment target is 154 participants, which with 20% dropout, is hoped to provide 124 people receiving treatment and follow-up.

**Discussion:**

This project introduces screening and brief interventions for high-risk drinkers admitted to the hospital with facial trauma. It introduces a practical approach to integrating brief interventions in the hospital setting, and has potential to demonstrate significant benefits for at-risk drinkers with facial trauma.

**Trial Registration:**

The trial has been registered in Australian New Zealand Clinical Trials Registry (ANZCTR) and Trial Registration: ACTRN12611000135910.

## Background

Globally, alcohol causes 3.2% of all deaths, or 1.8 million deaths annually, and accounts for 4.0% of disease burden [1]. Alcohol-related injuries are a problem in both high and low-income countries [2], including. Alcohol-related trauma is recognised as a major public health problem in Australia [3]. Alcohol abuse is a major contributor to the incidence of traumatic injury [4-7]: 27% to 47% of trauma patients test positive for alcohol use at the time of admission, and 30-40% test positive for other substance use [8]. Alcohol and other drug abuse induce physical and cognitive impairment that increases vulnerability to both unintentional injury and violence [9,10]. The impact of alcohol also extends to criminal offenses. An estimated 50% of all Australian offenders detained by police in 2007 for disorder and violent offences had consumed alcohol in the 48 hours prior to their arrest [11]. In 2004–2005, the cost of alcohol-related injury in Australia was estimated at AUD15.3 billion, when costs associated with crime and violence, treatment, loss of productivity and premature death were all included [12].

Australian Aboriginal and Torres Strait Islanders have high rates of injury, of hospitalisation and death in these people are caused by assault [13]. Alcohol represents a significant contributor to this increased risk [14].

The current research is conducted in Northern Territory (NT), which is situated in central and northern central Australia. It has a small population that is primarily located in two cities (Darwin and Alice Springs) and has extremely remote and sparsely populated areas. Between 2004 and 2009, the NT had the highest rate of per capita alcohol consumption in Australia (15 litres of ethanol) [15,16], and the highest estimated rate of alcohol-related hospitalisations for assault. The incidence of alcohol-attributable deaths in NT from 1990 to 2002 was 0.64 per 100,000 population, compared with 0.21 per 100,000 population nationwide [14]. The annual total cost to the NT from alcohol, tobacco and illicit drug abuse in 2009 was estimated at AUD642 million, or $4,197 per person [12].

The NT Aboriginal population is particularly at risk of alcohol-related harm and death, with 1.86 alcohol-attributable deaths per 100,000 people, compared with 0.38 in other NT residents [14]. Violence is the most common cause of hospital admission for injury in the NT, accounting for 38% of injury admissions for Aboriginal people [17]. Aboriginal prisoners are also vastly overrepresented in the NT, representing 82% (850) of the daily average prison population, but only 32% of the NT population [18]. Evidence of links between assault and alcohol misuse is scant, but reports from offenders clearly link alcohol in violent assaults and other crime [19].

A particular focus of alcohol-related violence involves mandibular fractures [20]. Facial fractures in the NT are close to 120 per 100,000 of population, and in Indigenous people occur at a massive 155 per 100,000 [21]. Personal assaults, fights, and violence account for 91% of all facial traumas in the NT [21]: 72% of these patients are injured by an intoxicated person when they were also intoxicated, and another 8% are by an intoxicated person when they were sober. Most assaults against women in remote NT communities are alcohol-related, and are perpetrated by a husband or other family member [22].

In the general population, screening and brief counselling can be effective in reducing alcohol intake and assault associated with binge drinking [23-26]. While there is abundant evidence that brief interventions are effective in the treatment of high-risk drinking [27], there is less research on the impact of brief interventions on alcohol-related violence [20].

The current project breaks new ground, in examining the impact of a brief inpatient intervention for alcohol-related facial trauma in a predominantly indigenous sample. In taking on this challenge, the cultural context of the intervention must be considered. A brief treatment that was specifically developed for use with indigenous Australians is Motivational Care Planning (MCP; Nagel et al. 2009). MCP incorporates key principles of several brief therapies: motivational interviewing, goal setting and problem solving. Motivational interviewing has been developed and used successfully as treatment for substance misuse and co morbidity with individuals and families [28-30]. In MCP, clients are encouraged to consider their life as a whole, rather than only focusing on the substance use, reviewing things that keep them strong, and take away their strength. A tree is used as a metaphor, and potentially affected domains (e.g. being on their land, spirituality, family) are presented pictorially. Among aspects that may take away strength is substance use, and clients are encouraged to consider the role this has in the overall picture.

Those who wish to make a behavioural change are encouraged to adopt a potentially achievable goal, and identify concrete steps toward it (represented as footsteps on a football field). Goal setting is well established as a strategy to guide effective self-management in a range of settings [31,32], and indigenous clients readily identify with the concept and metaphor adopted here. Potential issues are in achieving the initial steps are identified, and problem solving strategies are applied to these challenges [33]. In the current project, MCP is adapted to incorporate the nature of relationships between substance use and mandibular injury, while retaining a whole-of-life perspective.

## Aims of the study

The study aims to conduct a randomised controlled trial to compare the impact of a culturally adapted brief intervention (MCP) and of standard care, with patients hospitalised with alcohol-related trauma. The primary assessed outcomes include alcohol and other substance use and distress, which are examined at Baseline and 6 months. Incidence of further injuries is tracked, but the current study is not powered to detect differential changes in this index.

## Design

**Figure 1:**
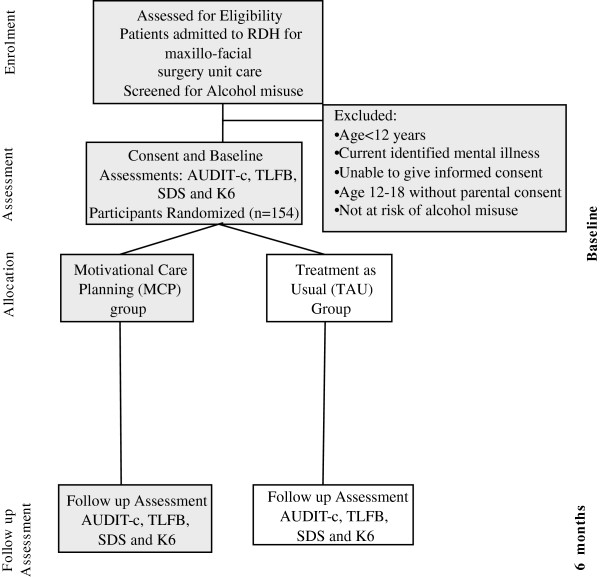
Expected CONSORT diagram.

This study is a parallel, randomised controlled trial to evaluate the effectiveness of a brief culturally adapted intervention in improving outcomes for high-risk drinkers admitted to hospital with facial trauma. The expected flow diagram for the study is shown in Figure 1.

## Recruitment

The study sample is opportunistically selected from patients who are admitted to the maxillofacial unit at Royal Darwin Hospital, in the Northern Territory of Australia. All at-risk drinkers admitted with facial trauma are assessed to determine their eligibility for inclusion. The hospital staffs assist the research team to identify potential participants.

## Inclusion/exclusion criteria

Inclusion and exclusion criteria ensure that the sample can engage with interventions and assessments [34].

All participants must satisfy the following criteria at study entry:

1. An inpatient of Royal Darwin Hospital with facial trauma.

2. At least 12 years of age.

3. Identified at risk of alcohol misuse as measured by the Alcohol Use Disorders Identification Test (AUDIT-C ≥ 6) [35,36].

4. Able to give informed consent (and if less than 18 years of age, parental consent is provided).

## Assessment

Assessments combine standardised measures and semi-structured interviews with individuals and family members who provide care or support. At Baseline, a demographic questionnaire gathers age, gender, location of residence in the NT, stressors protective factors including reviewing strengths, stressors, family and support networks and amount and frequency of substance misuse. Data is collected by trained Indigenous and non-Indigenous research officers and recorded on standardized forms. The screening and assessment tools have been presented in pictorial adaptation to the Aboriginal and Torres Strait Islander cultural and language context. The chosen measures (AUDIT-C, Kessler 6 [37]) have been tested in our previous work with Aboriginal and Torres Strait Islander people. They were found to be acceptable and are well understood.

### Screening instruments

#### AUDIT-C

The Alcohol Use Disorders Identification Test (AUDIT) is the quick estimate of alcohol consumption and designed to detect hazardous and harmful levels of alcohol consumption [38]. This gold-standard screening test is developed by the World Health Organization (WHO) as a simple method of screening for excessive drinking and to assist in brief assessment. The AUDIT is widely used as a universal screening tool for emergency department and primary health care patients in USA and UK [26,39]. The AUDIT has been used to assess risky alcohol use in an Indigenous population in Queensland, and has shown to perform well [40]. The AUDIT-C, a brief version of the AUDIT, consists of three items. This version has been shown to have similar sensitivity and specificity to the full questionnaire [41]. The third question of the AUDIT-C alone (which examines the frequency of respondents had 6 or more drinks) predicts alcohol-related morbidity [42]. Weekly binge drinking has high specificity (from 79% to 96%) and sensitivity (5% to 83%) [42-46]. Inclusion of this question in the current study is important, since consumption more than four drinks on a single occasion more than doubles the relative risk of an injury in the next 6 hours [47]. In the current study, data from the AUDIT-C are also used to assess alcohol consumption in cases where a full Timeline Followback measure (cf. below) cannot be obtained. The extent this abbreviated assessment is employed will be reported.

#### Severity of Dependence Scale (SDS)

*SDS* is a 5-item scale which focuses on the psychological aspects of dependence such as impaired control over drug use. It is a brief, easily administered instrument that is a reliable and valid screening tool in different cultural settings [48], in the context of dual diagnosis [49,50], and across different substances [51]. It is used to screen for presence of substance-related disorder in this study, using a cut off of 3 [51].

### Outcome measures

#### Timeline Follow back (TLFB)

The TLFB is a retrospective assessment of substance use, which employs recall of activities and events to cue estimates of consumption. Its use for assessment of alcohol consumption has been evaluated with clinical and non-clinical populations [52] and can generate precise information about patterns and variability [52]. In the current study, the TLFB is used to assess both alcohol and cannabis use over a 14-day period. After data is obtained on the 14-day period, participants are asked about whether that period was typical of their recent substance use. Estimates of their more typical use are recorded, and are subjected to secondary analyses.

#### Kessler distress scale

Kessler-6 (K6) is a 6-item version of the Kessler-10 (K10) measure of emotional distress [53]. The K10 is one of the consumer measures mandated for use in Australian mental health services, and has been validated among Australians with substance misuse [54]. The K6 is also highly predictive of mental disorder [55], showing a sensitivity of .85 and a specificity of .78 [56], compared with a sensitivity of 0.78 and specificity of 0.74 for the K10 [54]. Both the K10 and K6 have been used with Indigenous people in population surveys. In the current trial, K6 is used to assess psychological distress during the preceding month.

### Secondary outcome measures

File Audits: Hospitalisations for alcohol-related injuries and illness in the preceding two 6-month periods are determined from the patients’ files at Baseline and at 12 months post-treatment. In both cases, any mention alcohol being associated with an injury or illness is coded positive. We also code for any record of screening or assessment of alcohol use, distress or trauma over the period, and for any related intervention.

### Procedure

Eligible trauma patients are identified in the maxillofacial unit and referred to the research staff, who obtain informed consent. All eligible participants are screened for high risk drinking. Full assessment of those screened at risk is performed prior to random allocation. Those at risk are randomly allocated to Motivational Care Planning or Treatment as Usual conditions. A statistician who is not directly involved in the analysis of the study results prepares the randomisation code to ensure that an approximate balance between in numbers is maintained between groups throughout the study. Allocations are concealed until the person’s baseline assessment is completed. Sealed envelopes contain the sequences, and the use of the envelopes is monitored. Blinding and equipoise are strictly maintained through clear protocols, assessor training, and oversight of procedure by the Principal Investigator.

### Motivational Care Planning (MCP)

Elements of Motivational Care Planning are described above and in previous publications [57]. MCP was developed in collaboration with Aboriginal Mental Health Workers, and differs from established approaches by inclusion of pictorial tools and a holistic, family focus. The 30-minute intervention is manualised and is usually completed in a single session, and is delivered by both Indigenous and non-Indigenous mental health research staff. A non-Indigenous version of MCP is used for the non-Indigenous participants. It omits aspects of primary interest to Indigenous people (e.g. hunting and gathering).

### Treatment as Usual (TAU)

Participants randomised to TAU receive facial trauma treatment according to usual practice at the hospital, with addition of an information sheet on alcohol and trauma that was prepared in consultation with the project’s Expert Reference Group.

### Fidelity of the intervention

Therapists undertake a 2-day workshop on delivery of the manualised interventions, together with 3-monthly booster training and fortnightly supervision sessions to maintain fidelity. A sample of sessions are observed and rated by research investigators using a checklist of key features. Regular feedback on fidelity is given, with suggestions on how to adjust delivery.

### Follow up assessment

Face-to-face follow-up assessments are conducted at 6 months post-baseline, by researchers who are blind to condition. These assessments are conducted in the Royal Darwin Hospital, or in the closest health centre to the participant. In the case of people who cannot be contacted for face-to-face assessment, assessments are undertaken by telephone. Where participants cannot initially be contacted for follow-up assessments, attempts to contact them continue for up to 12 months post-baseline.

## Predictions

### Primary predictions

We predict that:

1. High-risk drinkers with maxillo-facial injuries who receive Motivational Care Planning will have greater reductions in (a) alcohol, on the TLFB and AUDIT-C; (b) other substance use on TLFB and (c) distress on K6, than participants receiving Treatment as Usual.

2. Greater improvements from Motivational Care Planning will be maintained to 6 months post-treatment.

#### Secondary predictions

Patients receiving Motivational Care Planning are expected to have reduced readmission rates for injury, although the study may be insufficiently powered to detect this effect.

## Sample size

Assuming equality between conditions at Baseline, the study is powered to detect a differential reduction in alcohol consumption of 0.50 SD in weekly alcohol consumption [58]. We argue that this is the minimum difference of any clinical significance. Setting the power at 0.80 and alpha at 0.05, 62 participants are required in each condition. While data will be analysed by intention to treat, the sample size allows for 20% attrition, deriving a target baseline sample of 154.

## Analysis

Continuous outcome variables are assessed for normality prior to analysis and transformed if necessary. Primary analyses use Linear Mixed Models analyses comparing the 2 conditions (MCP vs TAU) over 2 occasions (Baseline, 3 and 6 months post-baseline), allowing the analysis of data on all participants allocated to conditions at Baseline (i.e. intention to treat).

## Feasibility

A total of more than 250 patients are admitted to the Royal Darwin Hospital maxillofacial unit each year. We anticipate that 62% of these inpatients will fulfil eligibility criteria for inclusion and that 77 patients per year will be eligible for randomisation over the 12-month recruitment period.

In order to minimise drop out we will undertake the following actions:

•  Obtaining at least 3 means of contacting participants at recruitment (Telephone number of participant, telephone number their carers -family member and participants address);

•  Following up by phone to update contacts and residential details every month;

•  Liaising with the surgical team to link follow-up assessments with client outpatient visits.

## Consent and culturally appropriate approach

Research officers will use pictorial and plain English information sheets, screening and intervention tools to assist understanding. Aboriginal and Torres Strait Islander participants will be offered communication support (interpreters, translators, assessed communication and inclusion of a support and cross-cultural family member or person of their choice). All information will be presented to participants by Indigenous researchers in a culturally appropriate manner, and written assessments will be administered orally in cases of limited literacy.

## Ethics and confidentiality

The study has been granted ethical approval by the Human Research Ethics Committee of Department of Health and Families and Menzies School of Health Research. The approval number is HREC 2010–1438. Electronic data is password-protected, and identifying data is kept on a separate database from outcome data, allowing de-identification at the end of data collection. Assessment data will be accessible only to the investigators and support team.

## Discussion

This project will introduce a practical approach to integrating brief interventions into the hospital setting, and has the potential to demonstrate significant benefits for at-risk drinkers with facial trauma. Findings from this project are expected to inform hospital-based treatment and secondary prevention of alcohol-related injury, not just in indigenous people in NT, but in other trauma treatment units throughout the world.

## Competing interests

The authors declare that they have no competing interests.

## Authors’ contribution

JR conceived of the study, participated in its design and coordination, carried out the review, and drafted the manuscript. MT, DK, VT, CG, LM and Pd conceived of the study, participated in its design and helped draft the manuscript. TN conceived of the study, participated in its design and helped draft the manuscript. All authors read and approved the final manuscript.

## Pre-publication history

The pre-publication history for this paper can be accessed here:

http://www.biomedcentral.com/1472-6963/12/371/prepub
